# Identifying and understanding determinants of high healthcare costs for breast cancer: a quantile regression machine learning approach

**DOI:** 10.1186/s12913-020-05936-6

**Published:** 2020-11-23

**Authors:** Liangyuan Hu, Lihua Li, Jiayi Ji, Mark Sanderson

**Affiliations:** 1 Department of Population Health Science and Policy, Icahn School of Medicine at Mount Sinai, 1425 Madison Avenue, One Gustave L. Levy Place, Box 1077, New York, NY 10029 USA; 2 Department of Health System Design and Global Health, Icahn School of Medicine at Mount Sinai, New York, NY 10029 USA

**Keywords:** Medical care costs, Cancer, Machine learning, Quantile regression

## Abstract

**Background:**

To identify and rank the importance of key determinants of high medical expenses among breast cancer patients and to understand the underlying effects of these determinants.

**Methods:**

The Oncology Care Model (OCM) developed by the Center for Medicare & Medicaid Innovation were used. The OCM data provided to Mount Sinai on 2938 breast-cancer episodes included both baseline periods and three performance periods between Jan 1, 2012 and Jan 1, 2018. We included 11 variables representing information on treatment, demography and socio-economics status, in addition to episode expenditures. OCM data were collected from participating practices and payers. We applied a principled variable selection algorithm using a flexible tree-based machine learning technique, Quantile Regression Forests.

**Results:**

We found that the use of chemotherapy drugs (versus hormonal therapy) and interval of days without chemotherapy predominantly affected medical expenses among high-cost breast cancer patients. The second-tier major determinants were comorbidities and age. Receipt of surgery or radiation, geographically adjusted relative cost and insurance type were also identified as important high-cost drivers. These factors had disproportionally larger effects upon the high-cost patients.

**Conclusions:**

Data-driven machine learning methods provide insights into the underlying web of factors driving up the costs for breast cancer care management. Results from our study may help inform population health management initiatives and allow policymakers to develop tailored interventions to meet the needs of those high-cost patients and to avoid waste of scarce resource.

## Background

It is well known that healthcare costs are concentrated among a small group of ‘high-cost’ patients [1]. Despite they receive substantial care, many have unmet critical healthcare needs and receive unnecessary and ineffective treatments [2–5]. This suggests that ‘high-need, high-cost’ patients are a natural group to seek for healthcare quality improvement and cost reduction. In the US, providers and insurance plans have sought to develop care coordination and disease management programs to reduce hospital use and costs [6]. Research has shown that these programs are more effective when they are targeted to patients who most likely benefit [2, 7, 8]. Studies have looked into developing predictive models to identify high-cost patients prospectively [9]. Little is known, however, about the relative importance of clinical characteristics and demographic and social-economic status to the distribution of health expenditures. Identifying major underlying drivers of high healthcare costs and understanding how they are linked to different percentiles of the cost distribution, especially the upper tail where the medical expenditures are concentrated, will provide insights into designing effective and tailored interventions to meet the needs of high-cost patients and reduce costs.

Breast cancer diagnosis is the top cancer diagnosis among women in the US, accounting for 29% of all newly diagnosed female cancers each year [10]. The costs of breast cancer treatment and follow-up care put a strain on both healthcare system and patients. Cost of care in the first year after diagnosis varies from $54,664 to $127,444 depending on the stage at which breast cancer was diagnosed, based on the claim data from private insurers from 2003 to 2010 [11]. If measured by episode defined by the Oncology Care Model (OCM) – a payment model developed by the Center for Medicare & Medicaid Innovation (CMMI) – the total Medicare expenditure for breast cancer is $20,887 per episode on average, with the largest component chemotherapy accounting for 25.9% of the total spending [12].

The OCM is a new payment and delivery model that began on July 1, 2016 and runs through Jun 30, 2021. It is designed to improve the effectiveness and efficacy of specialty care. It aims to encourage participating practices to improve care and lower costs for Medicare fee-for-service beneficiaries with cancer through an episode-based payment model that financially incentivizes high-quality, coordinated care. The OCM collects rich information on episodes and patients from nearly 200 practices and 17 payers, including Center for Medicare & Medicaid Services (CMS), and is well suited for health services research. Since the main goal of the OCM is to set the target price so that performance of participating providers can be measured by comparing the actual cost to the target price, current research utilizing the OCM data generally focuses on expense prediction [13]. Investigating the underlying drivers of high costs for cancer care and how they affect high-cost patients is largely an untapped area [14]. In this article, we leverage the large number of episodes on breast cancer captured in the OCM data and establish the role of key drivers of high costs for breast cancer patients. We believe this is the first study to utilize the OCM data and aim to clarify the underlying drivers of high costs for cancer management.

**Fig. 1:**
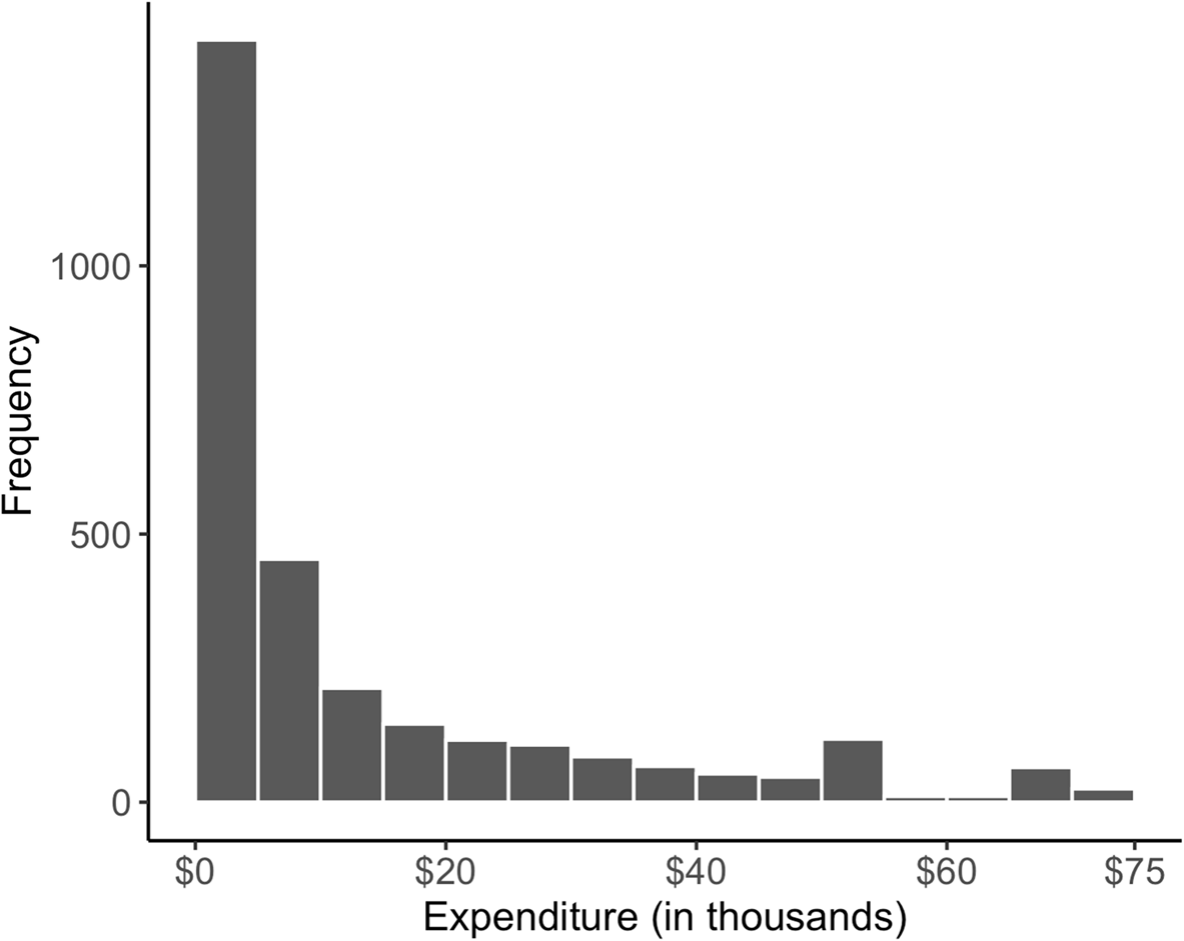
Histogram of episode based expenditures

Expenditure data is typically skewed and heteroscedastic. Figure 1 shows a histogram of OCM episode expenditures for breast cancer. The skewness measure is 1.67, indicating the expenditure distribution is highly skewed. Quantile regression (QR) methods are well suited to estimate how specified quantiles, or percentiles of the distribution of the outcome variable vary with covariates, and is robust against outliers and is more informative for a skewed distribution than mean-based regression [15]. We demonstrate the value of a highly flexible machine learning based quantile regression method in studying healthcare expenditures.

We used episode-based expenditure data on breast cancer, drawn from the OCM, and included 11 variables representing information on treatment, demography and socio-economics status. We then exploited quantile regression random forests (QRFs) – a machine learning modeling technique – to rank the relative importance of the covariates, and proposed and implemented a principled algorithm to identify a set of major determinants for high episode costs. We further quantified the effects of the identified major determinants on different quantiles of episode expenditures and emphasized new insights that can be gained relative to high cost patients.

## Methods

We extracted the cost and episode/patient related information from the data that OCM provided to Mount Sinai Hospital, which is a participating institution. The OCM is a voluntary 5-year episode-based payment program developed by the CMMI, which started in 2016 among 194 US oncology provider groups with the baseline period between January 2012 and June 2015. It was set to continue for 5 years, with the goal of improving care coordination and lowering care costs through episode-based cost performance and quality measures [16, 17].

The cost is arranged at the episode level. Each episode is triggered by either outpatient chemotherapy claim along with a corresponding cancer diagnosis on the claim, or the filling of a prescription for Part D covered chemotherapy [18]. The duration of an episode is 6 months from the triggering event or at the patient’s death. The eligibility criteria for a beneficiary’s episode to be included in OCM are: 1) beneficiary is enrolled in Medicare Parts A and B; 2) beneficiary does not receive the Medicare End Stage Renal Disease benefit; 3) beneficiary has Medicare as his or her primary payer; 4) beneficiary is not covered under Medicare Advantage or any other group health program; 5) beneficiary received chemotherapy treatment for cancer; 6) beneficiary has at least one qualifying Evaluation & Management visit during the 6 months of the episode. Episodes in which a beneficiary dies or elects hospice care before the end of 6 months are considered eligible; death will be the only case in which an episode will be shorter than 6 months [13]. The Mount Sinai OCM data included 2938 breast-cancer episodes from 1333 patients in both the baseline periods and three performance periods between Jan 1, 2012 and Jan 1, 2018 with the last episode ending on June 30, 2018. All the episodes were included in our analysis with no missing value.

We defined the actual cost associated with each episode as the outcome. It is the Medicare fee-for-service (FFS) expenditures incurred during each episode, which include all Medicare Part A and Part B FFS expenditures (which will include the OCM Monthly Enhanced Oncology Services payments), certain Part D expenditures, and payments resulting from overlapping participation in other Centers for Medicare & Medicaid Services models. We included 11 covariates used in the OCM risk adjustment model [13]. They were (1) Age, (2) Sex, (3) Chemotherapy drugs taken/administered during the episodes. It is grouped into two categorized: Part D (only Part D chemotherapy or long-term oral endocrine chemotherapy) such as tamoxifen and an aromatase inhibitor, and Part B (Part B chemotherapy or other therapies) such as antineo and cetuximab. The drugs included in each category can be found in the OCM therapy drug list provided by CMS [19]. Breast cancer episodes involving only part D or long-term oral endocrine chemotherapy tend to be much less costly than the episodes that involves other therapies [4]. Receipt of cancer-related surgery, [5] Part D eligibility and dual eligibility for Medicare and Medicaid, [6] Receipt of radiation therapy, [7] Clinical trial participation, [8] Comorbidities, which are measured through a subset of the CMS Hierarchical Condition Category (HCC) flags. These flags are created by CMS on a calendar year basis and indicate treatment for 70 different conditions in the prior calendar year. The number of HCC flags that are “turned on” indicates that episode expenditures increase with higher numbers of pre-existing comorbidities. Based on the number of HCC flags, we classify it into 6 categories: 0 flag, 1 flag, 2 flags, 3 flags, 4 flags and over, and new enrollee [9]. History of prior chemotherapy use, denoted by “clean period”. The clean period is calculated by the episode start date minus the date of the most recent chemotherapy claim before the episode start date and categorized into three category as in OCM: between 1 and 61 days; between 62 and 730 days; and 730 days above or no prior chemo claims [10]. Institutional status, indicating whether the beneficiary had been institutionalized in a long-term care facility for more than 90 days as of the month in which the episode started, and 11) Hospital Referral Region (HRR) relative cost, which captures the percentage difference in average episode costs between a given HRR and all HRRs. It is formulated as: HRR relative cost = [(Average episode cost for the HRR/Average episode cost across all HRRs) – 1] * 100. Based on this, a geographic adjustment will be made to distinguish episodes occurring in high- and low-cost areas.

**Fig. 2:**
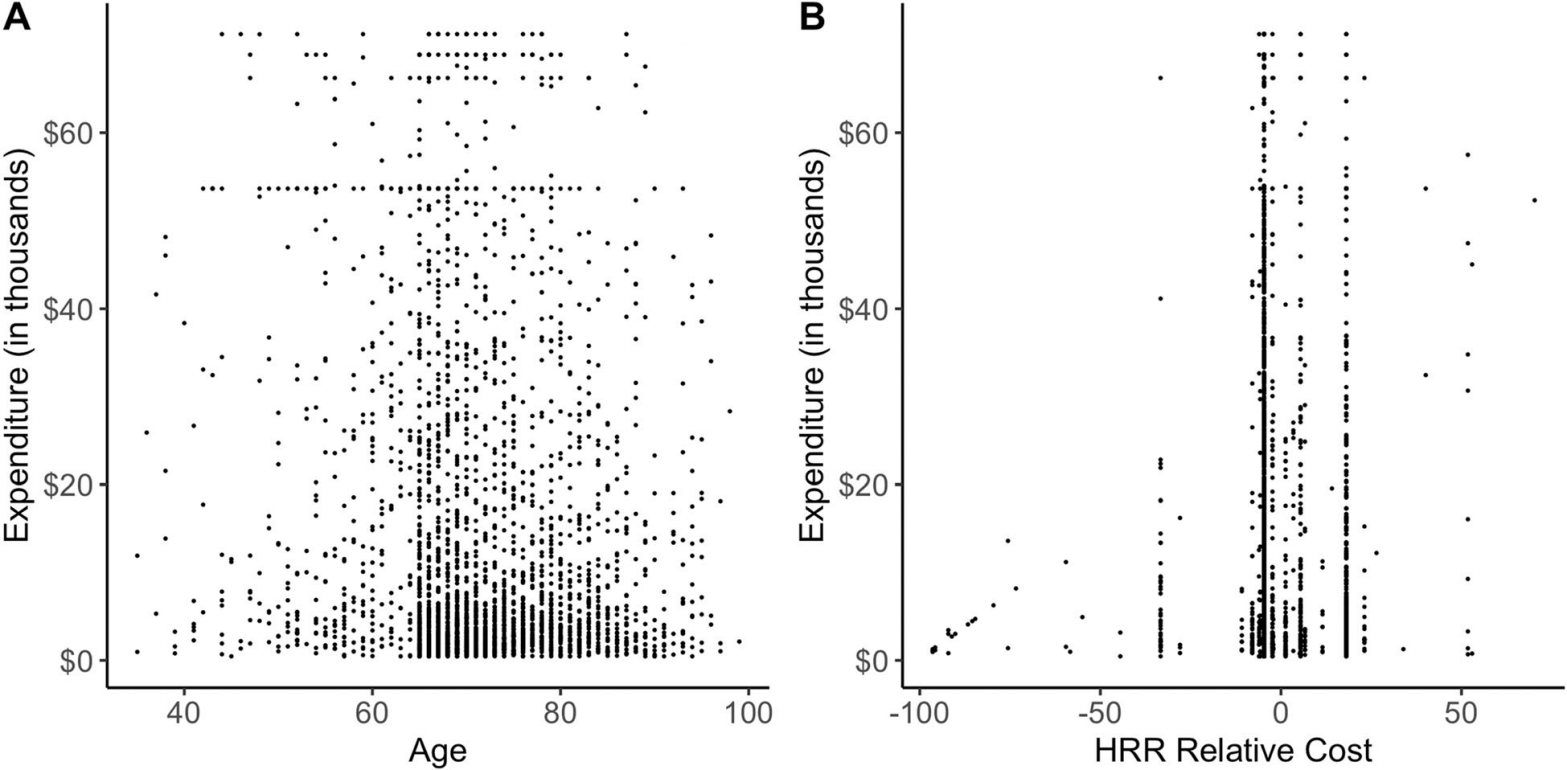
Scatterplots of episode expenditures versus age (A) and HRR relative cost (B)

The distribution of episode costs for each factor variable is summarized in Table 1, and scatterplots of episode costs for two continuous variables, age and HRR relative cost, are presented in Fig. 2. Our final analytical dataset included 2938 breast cancer episodes.

**Table 1:** Distribution of episode costs (in dollars) for each factor variable^e^

		Actual Episode Expenditures					
	N	Minimum	1st quartile	Median	Mean	3rd quartile	Maximum
**Sex**							
Female	2923	461.09	2272.80	5278.11	13,879.26	18,920.21	71,185.40
Male	15	1676.08	3378.14	5310.87	14,962.33	22,495.87	60,645.54
**Chemotherapy drugs**							
Part B	828	1486.41	17,582.50	30,595.09	33,026.14	48,364.65	71,185.40
Part D	2110	461.09	1766.81	3270.07	6373.39	6440.57	71,185.40
**Surgery**							
No	2818	461.09	2214.97	4949.55	13,449.74	18,088.10	71,185.40
Yes	120	1364.95	9037.30	17,372.43	24,101.19	35,220.38	71,185.40
**Insurance**							
No PartD^a^	127	1111.65	14,545.65	24,983.93	28,798.25	42,946.34	71,185.40
PartD LIS^b^	130	461.09	1989.09	4872.79	15,087.51	20,435.59	71,185.40
PartD NoLIS^c^	1794	461.09	1888.15	3828.62	11,102.19	12,297.31	71,185.40
Full dual^d^	887	461.09	3625.93	7926.81	17,201.15	24,179.71	71,185.40
**Radiation**							
No	2681	461.09	2154.71	4596.04	12,460.25	15,144.30	71,185.40
Yes	257	1393.47	12,220.82	25,329.08	28,745.46	42,778.24	71,185.40
**Trial participation**							
No	2923	461.09	2271.26	5260.65	13,869.41	18,975.22	71,185.40
Yes	15	2273.81	6065.91	16,671.52	16,880.54	18,742.96	48,306.45
**Comorbidities**							
0	828	461.09	1639.29	3450.09	11,750.76	14,716.46	71,185.40
1	727	461.09	2179.77	5079.50	12,080.17	14,285.17	71,185.40
2	469	461.09	2872.91	5500.16	14,587.20	20,551.60	71,185.40
3	288	461.09	2973.28	7702.07	16,603.32	24,587.09	71,185.40
≥ 4	271	469.21	5298.04	13,266.07	19,067.06	27,077.94	71,185.40
New enrollee	355	461.09	2026.10	5079.58	15,468.37	25,367.09	71,185.40
**Chemotherapy clean period**							
62–730 days	1235	461.09	1677.36	3147.19	6540.55	6463.20	71,185.40
> 731 days	657	461.09	2688.89	8603.03	16,839.45	25,329.08	71,185.40
1–61 days	1046	461.09	3957.41	12,421.50	20,700.20	33,603.99	71,185.40
**Institutional status**							
Yes	14	3515.99	7298.26	14,258.97	22,597.47	34,961.03	66,192.20
No	2924	461.09	2271.99	5260.37	13,843.07	18,831.37	71,185.40

Note: ^a^No PartD means no part D enrollment; ^b^PartD LIS means does not have full Medicaid benefits but does have Part D with low income subsidy; ^c^PartD NoLIS means has Part D enrollment but no low income subsidy; ^d^Full dual means full Medicaid benefits (including Part D and LIS). ^e^Age and HRR relative cost are continuous variables and not included in this table; scatterplots of episode costs versus age and HRR relative cost are shown in Fig. 2

We applied a nonparametric machine learning technique, QRFs, on the OCM expenditure data. QRFs extends the framework of the Random forests (RFs). RFs consists of an ensemble of classification and regression trees, each of which is learned from a bootstrapped sample via binary recursive splitting. The RFs is adept at capturing interactions and nonlinearities [20]. For its high prediction accuracy and adaptability, RFs has gained popularity in medical research [20–26]. QRFs uses the basis of RFs and gives an accurate way of estimating conditional quantiles (rather than the mean) for multivariate covariates [27]. QRFs grows an ensemble of regression trees as in the standard RF algorithm, but for each node in each tree, QRFs keeps the values of all observations in the node instead of just the means as in RFs. Using the entire distribution of the observations, QRFs can examine the effects of exposure for different quantiles and provide a fuller picture of the exposure-response relationship than mean-based RFs. For model validation, as the QRFs model performs prediction using the out-of-bag (OOB) observations – samples left out as the testing data in each decision tree construction, it can provide its own internal estimate of predictive performance that correlates well with either cross-validation estimates for test set estimates [28]. We also conducted a goodness-of-fit test of our QRFs model, using the metric *R1*, or 1 minus the ratio between the sum of absolute deviations in our QRFs models and the sum of absolute deviations in the null (non-conditional) quantile model [29].

**Fig. 3:**
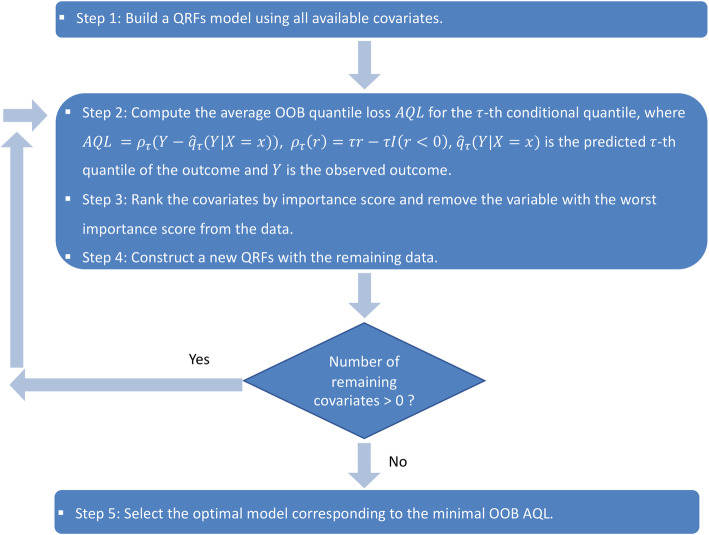
Quantile regression forests variable selection algorithm

We implemented a backward stepwise variable selection algorithm, which we previously developed, based on the variable importance scores generated by QRFs to determine the key factors for the 90th percentile of the episode expenditures [24]. The 90th percentile is commonly used in practice as the threshold for high-cost patients because the 10% of the population above the 90th percentile represents the group that incurred a disproportionately large share of all expenditures [9, 30]. The algorithm is summarized in Fig. 3. Details of the algorithm have been described elsewhere [24]. To obtain a reduced set of informative clinical characteristics associated with the upper tail of the episode costs, we implemented a backward stepwise QRFs. At each step, we removed the least important variable and rebuilt a QRFs model with the remaining variables and recorded the OOB average quantile loss (AQL) until no variable was left. AQL assesses the prediction error of *τ*-th (e.g., *τ* = 0.9) conditional quantile by averaging the quantile loss function over all observations [31, 32]. We identified the key determinants of the 90th percentile of the episode costs for breast cancer as the set of covariates corresponding to the QRFs model with the smallest AQL. Furthermore, we evaluated the relative importance of a variable by the reduction in AQL induced by the inclusion of that variable in the QRFs model.

Finally, to “unblackbox” machine learning, we included the major factors selected by QRFs in a classical linear QR model to quantify the effects of each factor on different quantiles of the episode expenditures. We used nature cubic splines with three degrees-of-freedom to model the smoothed effects of two continuous variables, age and HRR relative cost. All statistical analyses were performed using R version 3.6.1. QRFs models were built using the “quantregForest” R package.

## Results

**Fig. 4:**
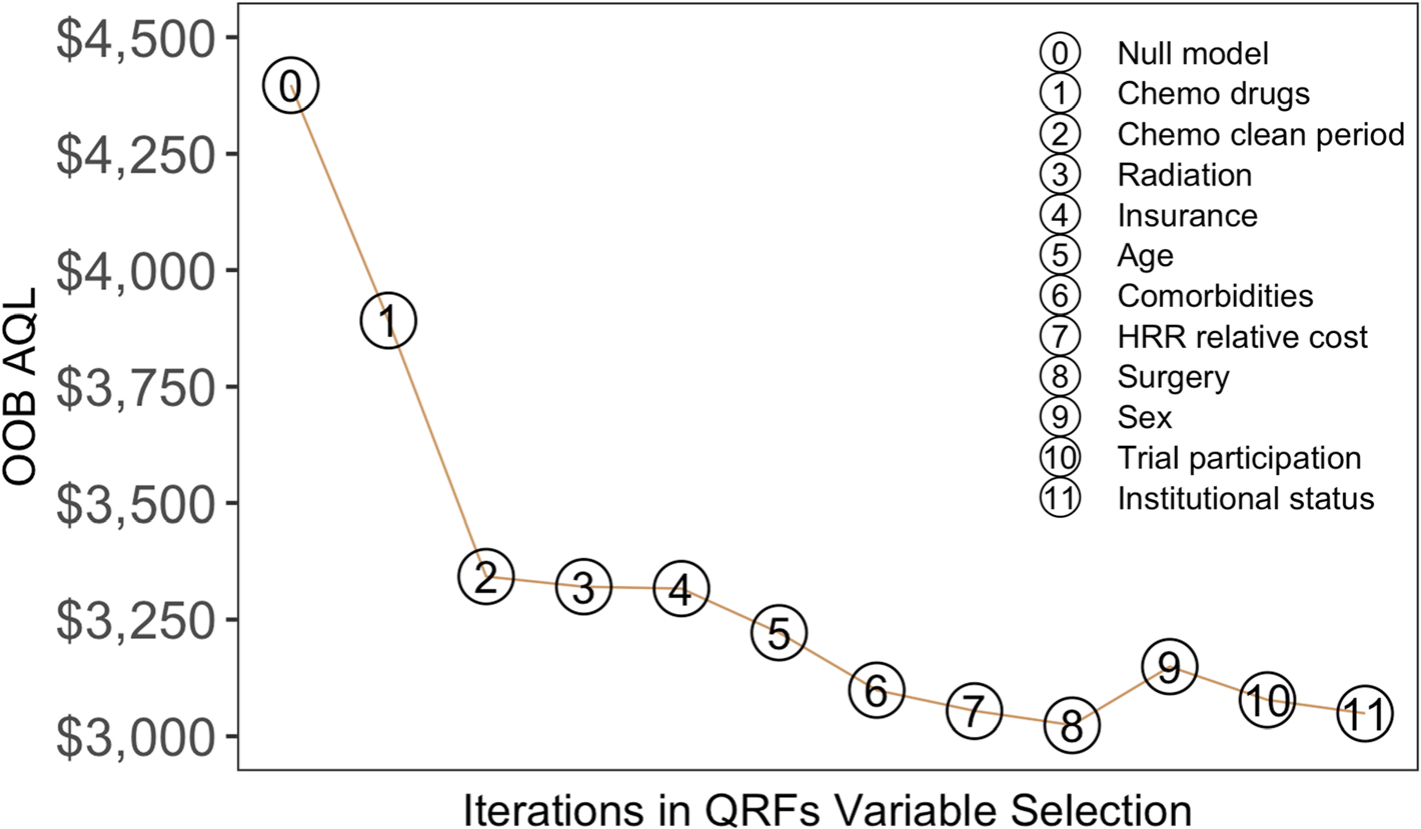
Estimated out-of-bag average quantile loss for the 90th percentile of episode expenditures corresponding to each QRFs model, which includes the remaining *k* variables (numbered by 1, 2, …, *k*) after sequentially removing variables (numbered by *k* + 1, …, 11) with lower importance scores, where *k* = 1, 2, …, 11. The null model is the intercept only model

Figure 4 shows, for the 90th percentile, the estimated OOB AQL for each QRFs model built at each iteration of our stepwise backward algorithm. The “optimal” QRFs model with the smallest prediction error suggests eight determinants for the upper tail of the cost distribution, including chemotherapy drugs used or administered, chemotherapy clean period, radiation therapy, eligibility for Medicare and Medicaid, age, comorbidities, HRR relative cost and surgery. The goodness-of-fit test of our QRFs model for the 90th percentile was 0.78, indicating a reasonably good model fit.

The relative importance of each variable is also implied in Fig. 4. Higher numbered variables were removed from the QRFs model earlier than lower numbered variables. The drop in AQL induced by the inclusion of a variable implies the importance of that variable to the outcome. Taken together, chemotherapy drugs used or administered during episodes and chemotherapy clean period were two predominant factors of the 90th percentile of the episode expenditure; they jointly accounted for 77% of the total reduction in AQL from the null model (with no covariates) versus the optimal model (with eight key determinants).

We further provided an “unblackboxing” analysis to quantify the effects of the identified key factors on the episode expenditures. To demonstrate that a variable may have different effects across quantiles of the outcome distribution, we examined the respective effects on the 90th (upper tail), 75th, 50th (median), 25th and 10th (lower tail) quantile. To explore the possible nonlinear age effects, we also fitted a separate model using nature cubic splines with three degrees-of-freedom to capture the smoothed effects of age.

Table 2 summarizes the point estimates and 95% confidence intervals for each of the eight major determinants. First, compared to long-term hormone therapy, other non-chemotherapy drugs, Part B drugs and Part D drugs were all associated with higher costs across all percentiles of the cost distribution. Manifested by the largest effect estimates, Part B drugs were the most expensive drugs for breast cancer. Both short (1–61 days) and long (> 730 days) periods of no chemotherapy were linked to higher costs among high-cost patients compared to the quiescent phase of treatment (clean period of 61–730 days), suggesting a “U” shape with highest costs at onset of disease and at death [33]. Radiation, surgery and multimorbidity were all associated with higher costs across different quantiles. While full medication insurance in general incurred higher costs than other partial insurance types, eligibility for Medicare and Medicaid was only associated with median costs with inconclusive effect on other percentiles of the cost distribution. There was a strong association between HRR relative cost and the episode expenditures among high-cost patients, suggested by much higher effect -- every 30 units increase in HRR relative cost was associated with $1800 (95% CI, $1000, $2600) higher expenditure -- for the 90th quantile than for the 10th quantile -- every 30 units increase in HRR relative cost was associated with $100 (95% CI, 0, $200) higher cost. This finding is consistent with previous study findings that individuals living in the high cost area go on to use more hospital resources [34]. With age, on average, there was a decreasing trend showing that older patients were associated with less episode expenditures; and this trend was more evident among the high cost patients (e.g., 90th percentile) compared to low cost patients (e.g., 10th quantile).

**Table 2:** The effects (point estimate [95% confidence interval]) of eight major factor variables on episode expenditures varied across the 10th, 25th, 50th, 75th and 90th quantile of the expenditure distribution. Effects are measured in thousands of dollars

	10th quantile	25th quantile	50th quantile	75th quantile	90th quantile
(Intercept)	−4.5 (−14.4, − 1.6)	−3.3 (−6.6, − 1.4)	−3.2 (−7.7, 2.4)	3.1 (−3.6, 14.0)	8.7 (−7.1, 15.9)
**Chemotherapy drugs (ref = Part D) (*n*** **= 2110)**					
Part B (*n* = 828)	9.5 (8.1, 10.9)	16.3 (14.0, 18.6)	25.0 (23.0, 27.1)	40.6 (37.7, 43.5)	47.8 (43.9, 51.7)
**Chemotherapy clean period (days, ref = 62–730) (*n*** **= 1235)**					
> 730 (*n* = 657)	0.1 (− 0.2, 0.2)	0.3 (0.1, 0.5)	0.6 (0.2, 0.9)	1.5 (0.5, 2.4)	3.7 (1.6, 5.5)
1–61 (*n* = 1046)	0.1 (0.0, 0.3)	0.5 (0.3, 0.7)	0.8 (0.4, 1.3)	1.7 (0.9, 2.7)	6.2 (3.3, 9.6)
**Radiation (ref = No) (*n*** **= 2681)**					
Yes (*n* = 257)	3.8 (2.5, 4.9)	6.4 (4.5, 7.3)	7.2 (5.8, 8.8)	7.1 (5.2, 10.3)	9.2 (5.2, 13.6)
**Insurance (ref = No PartD**^a^**) (*n*** **= 127)**					
PartD LIS^b^ (*n* = 130)	2.4 (− 0.8, 4.9)	2.7 (− 0.2, 5.8)	4.3 (1.7, 9.3)	3.1 (− 0.5, 10.4)	0.7 (−3.4, 13.1)
PartD NoLIS^c^ (*n* = 1794)	2.7 (− 0.6, 5.7)	2.9 (− 0.2, 6.0)	3.6 (0.3, 8.3)	0.8 (− 2.5, 9.3)	− 0.8 (−4.1, 5.3)
Full dual^d^ (*n* = 887)	3.2 (0.1, 6.1)	3.9 (0.9, 7.0)	5.2 (2.0, 10.1)	4.3 (0.8, 12.9)	2.8 (−0.1, 10.3)
**Comorbidities (ref = 0) (n = 828)**					
1 (*n* = 727)	0.2 (0.1, 0.3)	0.3 (0.1, 0.5)	0.5 (0.3, 0.8)	0.8 (0.1, 1.6)	0.5 (−1.2, 2.1)
2 (*n* = 469)	0.6 (0.3, 0.9)	1.0 (0.7, 1.3)	1.3 (1.0, 1.7)	1.8 (1.1, 3.0)	1.5 (0.3, 3.1)
3 (*n* = 288)	0.8 (0.5, 1.1)	1.3 (0.8, 1.6)	1.5 (1.0, 2.2)	3.2 (1.1, 4.6)	3.3 (1.3, 7.5)
≥ 4 (*n* = 271)	1.4 (0.9, 1.8)	1.7 (1.2, 2.3)	3.1 (2.2, 4.8)	4.2 (2.5, 5.7)	6.2 (4.3, 7.8)
New enrollee (*n* = 355)	0.0 (−0.4, 0.3)	0.4 (−0.1, 0.8)	0.4 (0.1, 0.9)	−0.2 (−1.1, 0.8)	1.2 (− 1.0, 4.6)
**HRR relative cost (in 30)**	0.1 (0.0, 0.2)	0.4 (0.1, 0.7)	0.8 (0.4, 1.2)	1.2 (0.8, 1.6)	1.8 (1.0, 2.7)
**Age (10 years)**	−0.1 (− 0.2, 0.0)	−0.2 (− 0.3, 0.1)	−0.7 (−1.1, − 0.3)	− 1.2 (− 1.7, − 0.8)	− 1.8 (− 2.2, − 1.2)
**Surgery (ref = No) (*n*** **= 2818)**					
Yes (*n* = 120)	5.2 (3.7, 6.5)	5.6 (4.7, 6.4)	6.7 (5.1, 7.9)	6.8 (4.7, 10.3)	8.6 (5.0, 13.8)

Note: ^a^No PartD means no part D enrollment for prescription drug coverage; ^b^PartD LIS means does not have full Medicaid benefits but does have Part D with low income subsidy; ^c^PartD NoLIS means has Part D enrollment but no low income subsidy; ^d^Full dual means full Medicaid benefits (including Part D and LIS)

**Fig. 5:**
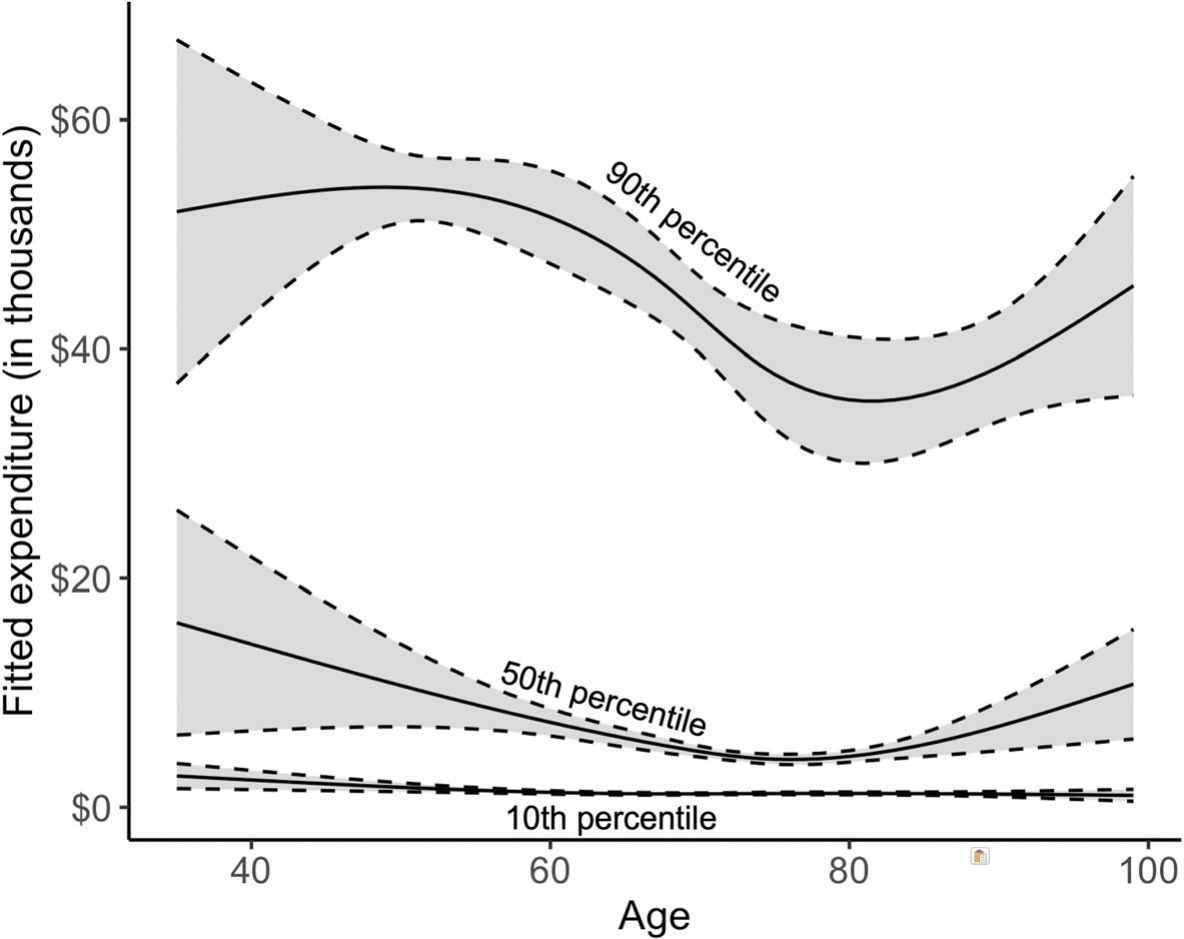
Effect estimates of age on the 10th, 50th and 90th quantile of the episode cost distribution, using natural cubic splines. To obtain sufficient legibility, we did not plot results for the 25th and 75th quantile

The fitted splines of age in Fig. 5 suggest their nonlinear effects on the costs. The 90th percentile of the costs was highest among patients aged 50–55, then gradually decreased through age 80 before turning up towards the end of life.

Second, our results demonstrate that the effects of the eight determinants upon episode-based expenditures are not uniform, but are in general disproportionally larger on the right tail of the cost distribution, i.e., those who already have the highest expenditures. For example, compared to long-term hormone therapy, Part B chemotherapy drugs cost $53,800 (95% CI, $49,900 – $56,800) more among high-cost patients (sitting at the right tail) and $10,200 more among low-cost patients (sitting at the left tail). Compared to the quiescent period with a chemotherapy clean interval of 62–730 days, a clean interval of less than 61 days (e.g., around the onset of disease) cost $6300 more among high-cost patients and only $100 more among low-cost patients. These findings suggest that our QR based analyses provide a full picture about the effects of exposures. For HRR relative cost for example, the effect of HRR relative cost is negligible among low cost patients (10th percentile) but is markedly evident among high cost patients (90th percentile).

## Discussion

In this study, we applied a robust and reproducible machine learning based approach to identify major factors for high-cost breast cancer patients, when the cost distribution was highly skewed, and investigated the underlying effect mechanisms of the major factors, leveraging a high-performance nonparametric quantile regression technique, QRFs. We exploited a Mount Sinai OCM cost data set on nearly 3000 breast cancer patients with episode-based clinical information and demographic and social-economic status.

Our results provided insights into drivers of high medical costs for breast cancer. Our approach identified eight determinants that jointly impact episode-based expenditures for breast cancer among high-cost patients. Among these factors, chemo drugs and chemo clean period were two predominantly influential variables, followed by the number of comorbidities and age. These determinants did not uniformly impact upon the expenditures, but disproportionally affected the high-cost patients, and their effects on low-cost patients may be negligible. Using mean-based methods would have ignored the disproportionality in the effect estimates, leading to a limited and biased conclusion. Our approach offered a “higher-resolution” analysis that can be used to expand and deepen the existing quantitative evidence on clinical risk factors for episodes expenditure.

Results from our study may help inform population health management initiatives. Establishing key determinants for high-cost cancer patients allows policymakers to develop tailored interventions to meet the needs of those high-cost patients and to reduce high cancer costs. For example, among those who are already high cost patients, the age cohort 50–55 was found to be associated with the highest costs. Developing strategies to reduce care spending tailored for this age cohort may help avoid waste of scarce resource. The Part B chemotherapy drugs, a chemotherapy clean interval of less than 61 days and multimorbidity were all drivers of high costs among those who already had the highest spending. These findings may provide insights into strategies for expanding the scope of care management programs investigating preventable spending. Currently such programs are relatively narrow and could have included more broad measures of preventable or wasteful spending [6]. Our results may assist in developing algorithms targeted at subgroups defined by identified underlying high-cost drivers to avoid preventable costs through interventions such as reducing duplicate services, contraindicated care, unnecessary laboratory testing or prolonged hospitalizations [6].

There are several limitations in this study. First, we the relationships between clinical and health characteristics and medical costs do not bear a causal interpretation due to the nature of the cross-sectional data [35–37]. However, our results identified important factors of high costs for breast cancer and can potentially stimulate future causal inference research in cost analysis. Second, the cost data for this study, made available by CMS, has both pros and cons. This single payer data allows for a comprehensive, consistent dataset that includes all of the health care services provisioned for a patient. However, it is limited to an elderly population and may not be reflect spend drivers for commercial members [38]. Also, the Medicare dataset included Medicare payments only, and did not incorporate out of pocket expenses which can be significant for medications in Part D. Third, our data is from a single institution. Despite the lack of national representation, because the Mount Sinai Hospital is one of the nation’s largest hospitals, we were able to include a large number of episodes in our analysis. Our methods are highly flexible and reproducible, and can be applied to a larger set of OCM data for breast cancer or other data sets alike for other kinds of cancer. Finally, there could be other important variables that were not included in our study, either unmeasured or not collected in our data, such as the accurate capture of disease progression [39]. Though the type of drugs at some level reflects the disease severity, cancer stage is not collected in the OCM data. CMS is working to expand the factors of the OCM to consider disease progression. Developing a sensitivity analysis strategy to evaluate the impact of unobserved data could be a worthwhile contribution [40]. Despite the potential omitted variables, by using an innovative and principled machine learning approach on a high-quality dataset with sufficiently large sample size, we believe the scope and depth of our analysis can provide important insights on policymaking and lead to more innovative investigations in the area of breast cancer health services research.

Uncovering true underlying determinants and their relative importance is challenging, especially when the exposure-outcome relationship may be nonlinear and the outcome is heavily skewed.

In public health research, determinants are often selected a priori or using test procedures based on some arbitrary threshold value. On the other hand, many cost analyses focus on building predictive models to identify high-cost patients. It remains unclear how the underlying complex web of factors drive up the costs for breast cancer. Our method is highly agnostic, leveraging flexible machine learning, and provides “higher-resolution” analysis for specific insights into important drivers for high costs and the detailed effect mechanisms on the costs among patients with varied level of costs. In conjunction with the relative importance of determinants, our method can provide valuable guidance for tailored and effective high-cost prevention interventions.

## Conclusions

High-performance and data-driven machine learning methods provide insights into the underlying web of factors driving up the costs for breast cancer care management. Results from our study may help inform population health management initiatives and allow policymakers to develop tailored interventions to meet the needs of those high-cost patients and to avoid waste of scarce resource.

## Data Availability

The OCM data that support the findings of this study are available from Center for Medicare & Medicaid Innovation but restrictions apply to the availability of these data, which were used under license for the current study, and so are not publicly available. Data are however available from the authors upon reasonable request and with permission of Center for Medicare & Medicaid Innovation. Analysis R codes are available from the corresponding author.

## Abbreviations

OCM: Oncology Care Model CM
CMS: Centers of Medicare &Medicaid Services
CMMI: Center for Medicare and Medicaid Innovations
QRFs: Quantile regression random forests
RFs: Random forests
QR: Quantile regression
AQL: Average quantile loss
OOB: Out of bag
HRR: Hospital referral region
FFS: Fee-for-service
HCC: Hierarchical condition category
